# Stability-based approaches in chemoproteomics

**DOI:** 10.1017/erm.2024.6

**Published:** 2024-04-12

**Authors:** Amy L. George, Maria Emilia Dueñas, José Luis Marín-Rubio, Matthias Trost

**Affiliations:** Laboratory for Biomedical Mass Spectrometry, Biosciences Institute, Newcastle University, Newcastle-upon-Tyne, NE2 4HH, UK

**Keywords:** chemical proteomics, chemoproteomics, drug discovery, limited proteolysis, mass spectrometry, proteome integral solubility alteration, target deconvolution, target engagement, thermal proteome profiling

## Abstract

Target deconvolution can help understand how compounds exert therapeutic effects and can accelerate drug discovery by helping optimise safety and efficacy, revealing mechanisms of action, anticipate off-target effects and identifying opportunities for therapeutic expansion. Chemoproteomics, a combination of chemical biology with mass spectrometry has transformed target deconvolution. This review discusses modification-free chemoproteomic approaches that leverage the change in protein thermodynamics induced by small molecule ligand binding. Unlike modification-based methods relying on enriching specific protein targets, these approaches offer proteome-wide evaluations, driven by advancements in mass spectrometry sensitivity, increasing proteome coverage and quantitation methods. Advances in methods based on denaturation/precipitation by thermal or chemical denaturation, or by protease degradation are evaluated, emphasising the evolving landscape of chemoproteomics and its potential impact on future drug-development strategies.

## Introduction

Phenotypic drug screening has regained traction in drug discovery, wherein compounds undergo assessment within a relevant biological model to induce specific molecular phenotypes (Ref. 1). Evaluating the overall effects of treatments on cellular behaviour provides a holistic view, leading to the discovery of many first-in-class compounds without any prior knowledge of protein targets (Ref. 2). This stands in contrast to target-centric approaches that focus on specific proteins (Ref. 3). However, to understand how these hit compounds exert their therapeutic effects, subsequent target deconvolution is necessary. Knowledge of these proteins targets is important for optimising compound safety and efficacy, elucidating mechanism of action (Ref. 4), identifying potential off-target proteins to anticipate side effects (Refs 5, 6, 7), and recognising opportunities for therapeutic expansion (Refs 8, 9). For instance, the identification of additional protein targets has facilitated the repurposing of existing drugs with established safety profiles for alternative diseases (Refs 1, 10).

The coupling of chemical biology with mass spectrometry, known as chemoproteomics, has revolutionised target deconvolution capabilities. Classically, small molecules can undergo covalent modifications by being immobilised, tagged or labelled with probes to ‘capture’ interacting proteins, producing an enriched sample for proteomic analysis (Ref. 8). While these methods such as affinity-based target identification and activity-based protein profiling have been extensively employed and previously reviewed (Ref. 11), these target-enrichment approaches may not be universally applicable to all small molecule compounds and could potentially impact the bioactivity or binding specificity of the drug (Ref. 8).

Herein, we will discuss recent modification-free approaches that leverage the direct stabilisation induced by small molecule ligands. The interaction of a drug and protein can alter its biophysical properties, influencing its resistance against thermal and chemical denaturation or enzymatic degradation. These changes can be identified as distinctive differences in soluble protein abundance or proteolytic digestion patterns under stress conditions in the presence and absence of the drug, on a proteome-wide scale. Although each method differs in workflow, applicability across experimental models and quantitative approaches (Table 1), all these methods have experienced substantial improvements in sensitivity and expanded proteome coverage due to the significant advancements in mass spectrometry observed over the last decade. Notably, the emergence of isobaric labelling technology such as tandem mass tags (TMT) has facilitated the multiplexed comparison of different conditions (Refs 5, 12). Additionally, the remarkable progress in data-independent acquisition (DIA) has found utility in some specific approaches, providing excellent proteomic coverage (Ref. 13).

**Table 1.** Key examples of label-free chemoproteomics approaches: strengths and limitations

Method	Advantage(s)	Limitation(s)	Model	Proteome coverage	Reference
Thermal denaturation					
TPP-TR	Identify targets, off-targets or downstream effectors Physiologically relevant environment	Variable High sample number, labour intensive False negatives as relies on curve fitting Not applicable to thermally stable proteins Multi-TMT batch required	Live cells Lysates	7500	(Ref. 22)
TPP-CCR	Identify targets, off-targets or downstream effectors Physiologically relevant environment Provide further information on drug efficacy	Variable High sample number Multi-TMT batch required Not applicable to thermally stable proteins	Live cells Lysates	7500	(Ref. 22)
2D-TPP	Identify targets, off-targets or downstream effectors Physiologically relevant environment Provide further information on drug efficacy Eliminates false positive hits by monitoring dose-dependent protein stabilisations	Variable High sample number Multi-TMT batch required False negatives Not applicable to thermally stable proteins	Live cells Lysates	8500	(Ref. 5)
iTSA	Increased statistical power with more replicates Higher throughput	Simplified experimental design Bias against proteins with higher or lower melting temperatures deviating from the median *T*_m_	Live cells Lysates	6500	(Ref. 50)
Proteolytic degradation					
DARTS	Can be used for target/off-target identification as independent of any effects of the drug on the system Enrich for target proteins, depleting non-target proteins	Measures abundant proteins with visible difference on gel (in-gel digestion only) Ineffective for studying stress proteins as tolerant to protease digestion False negatives	Lysates	<1000	(Ref. 55)
LiP-MS	Can be used for target/off-target identification Provides site-specific information with high resolution	Requires high sequence coverage and accurate quantification Conformational change may hamper binding region identification Not all small-molecule binding affects protease susceptibility	Lysates	6000	(Ref. 13)
Chemical denaturation					
SPROX	Can be used for target/off-target identification as independent of any effects of the drug on the system	Limited to methionine-containing peptides Lots of starting material required Not all methionine residues exhibit differential oxidation rates	Lysates	3000	(Ref. 77)
SPP	Complementary approach to TPP	Relies on generation of complete curves Substantial number of samples, labour intensive Multi-TMT batch required	Lysates	8000	(Ref. 85)
Protein solubility					
PISA	Increased statistical power Higher throughput No need for curve fitting Within TMT-batch analysis Identify targets, off-targets or downstream effectors Physiologically relevant environment Compatible with samples with lower protein amount	No additional information from curve generation	Live cells Lysates	8000	(Ref. 88)

In this review, we provide an update on methodological advancements, discussing the strengths and limitations of each approach in target deconvolution for drug discovery. A comprehensive understanding of these differences is crucial when selecting the appropriate chemoproteomic technique for drug–protein interaction studies.

## Target deconvolution based on thermal denaturation

### Thermal proteome profiling

When proteins are exposed to heat, the hydrogen bonds and hydrophobic interactions supporting their folded structure are disrupted, causing a transition into an unfolded state. This exposes their hydrophobic core, resulting in an insoluble precipitation (Ref. 14). The energy required to disrupt protein structure is altered upon ligand binding, causing a generally higher shift in melting temperature (*T*_m_) as energy is also required to dissociate the bound ligand (Refs 15, 16). This phenomenon has been used in drug discovery for assessing target engagement of purified proteins (Ref. 17). In 2013 it was applied in the cellular thermal shift assay (CETSA) to directly probe target engagement in complex biological samples, including inside live cells, for the first time (Ref. 18). Cell lysates, intact cells or tissues are treated with a compound and vehicle control, allowing sufficient time for protein–drug binding while limiting influence on protein expression. Subsequently, samples are aliquot and heated at increasing temperatures. Denatured protein precipitates are removed by centrifugation, and the remaining soluble fraction is collected for measurement of target protein stability using Western blotting or microtiter-based antibody assays (Refs 18, 19, 20). In addition, drugs can be assessed over an increasing concentration at a single temperature, known as isothermal dose–response (ITDR), in both cell and lysates to assess levels of target engagement and provide further information on drug efficacy (Refs 18, 20).

The principles of CETSA were coupled with multiplexed quantitative mass spectrometry, called thermal proteome profiling (TPP), extending from assessment of thermal stability in predefined targets to enabling proteome-wide investigation to identify unknown drug targets or off-targets causing toxic side-effects, and monitor downstream effectors (Refs 21, 22). In live cells, TPP provides additional information on downstream effectors that are dependent on the cellular environment (Ref. 16). Binding of the primary protein target may result in downstream effectors also undergoing changes in thermal stability, as protein–protein interactions (PPI) and altered post-translational modification (PTM) can also induce changes in protein stability (Ref. 16). These effects can be distinguished by performing TPP in lysates without functional cellular machinery (Refs 16, 21, 22). TPP has been applied to study PPI (Refs 23, 24), PTM (Refs 25, 26, 27, 28, 29), mutation (Ref. 30) and proteoforms (Ref. 31), protein function and cellular processes' (Refs 32, 33) in various sample types (Refs 34, 35) and biological systems (Refs 29, 36, 37).

**Figure 1.**
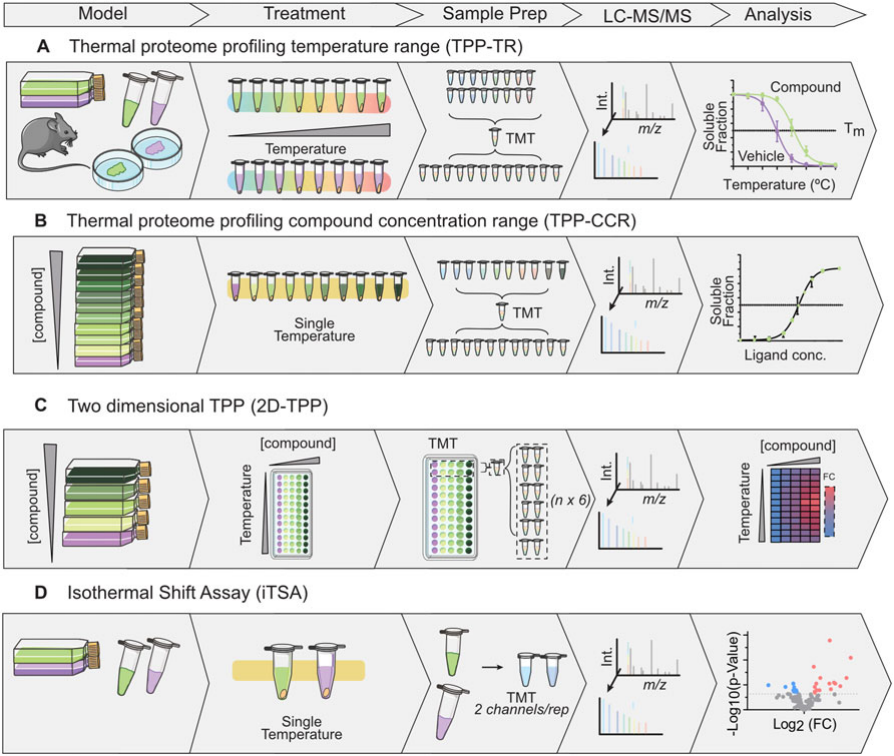
Schematic representation of chemoproteomic approaches based on thermal denaturation. All of these methods are technically applicable to cell lysates, live cells and tissues. (A) TPP-TR: samples are treated with a single dose of compound and subject to an increasing temperature gradient. Protein abundance in soluble fraction of each temperature labelled with TMT, pooled and fractionated. Melting curves constructed in the presence and absence of the drug are used to calculate shift in melting point (*T*_m_). (B) TPP-CCR: cells are treated with different compound concentrations and a vehicle control at a fixed temperature, generating affinity data. (C) 2D-TPP: dose-dependent thermal stabilisation profiling. (D) Isothermal shift assay (iTSA) measures difference in the soluble protein fraction at a single temperature, requiring only two TMT channels per biological replicate. By freeing up available TMT channels, more replicates can be condensed into a single experiment as fractionation is still performed. This leads to a four-fold increase in throughput, allowing for more replicate analyses, which improves statistical power.

In temperature range TPP (TPP-TR) (Fig. 1A), insoluble protein is removed by ultracentrifugation (100 000 × *g*) to improve signal-to-noise ratio (Ref. 21), or more recently with filter-aided benchtop centrifugation methods that increased throughput (Refs 37, 38). Each temperature point in the vehicle and drug-treated conditions is labelled with a different isobaric TMT for precise and multiplexed relative quantification. Originally, TMT-10plex was recommended to label 10 temperatures for melting curve generation, requiring two batches per biological replicate for both treatment and control conditions (Refs 21, 22). However, with the development of TMTpro 16-plex and 18-plex, it became feasible to use up to nine temperatures for each melting curve generation (Ref. 12). Samples are pooled, fractionated and analysed together in a single liquid chromatography–mass spectrometry (LC–MS) experiment. This improved quantitative precision and sensitivity by reducing technical variation and facilitating direct comparisons within the same experiment (Ref. 12). By plotting the relationship between temperature and soluble protein abundance, a sigmoidal melting curve is generated for each protein under a particular condition, enabling the calculation of the melting point (*T*_m_). The *T*_m_ represents the temperature at which 50% of the protein has denatured, and significant shifts in thermal stability due to ligand binding can be identified using the TPP package (Refs 21, 22).

Notably, the size of thermal shift does not directly depend on the affinity of the ligand but is unique to the protein's thermodynamics (Ref. 16). Therefore, the concentration of drug causing 50% of the total stabilising effect can be calculated using TPP-compound concentration range (TPP–CCR) (Fig. 1B), where cells are treated with nine different compound concentrations, and a vehicle control (using TMT10 reagents) at a fixed temperature (Refs 21, 22). This can generate affinity data similar to ITDR-CETSA, allowing better comparison of binding affinities among multiple protein targets and comparison of potency across different compounds (Refs 21, 22). These two approaches were soon combined by two-dimensional TPP (2D-TPP) (Fig. 1C), where cells are incubated with five varying compound concentrations while subjecting each sample to 12 incremental temperature changes across 6 multiplexed TMT10 experiments (Refs 5, 39). This approach not only provides valuable insights into compound-target affinity but also eliminates false positive hits by monitoring dose-dependent protein stabilisations (Ref. 16).

TPP traditionally involves fitting of sigmoidal curves to calculate melting temperature in vehicle and treated samples and uses this single parameter to assess for shifts in protein thermal stability (Refs 5, 21, 22, 40, 41). However, this analysis approach suffers from a high rate of false-negatives as the filtering based on curve quality-parameters for calculating *T*_m_ are extremely stringent and has limited statistical power (Refs 42, 43). A substantial portion of the proteome can provide poor fitting curves as they do not establish sigmoidal melting profiles, resulting in their exclusion from statistical analysis. For example, proteins may exist in multiple transitions (Refs 18, 22), ligand-binding does not always cause aggregation (Ref. 18), the temperatures used in a given experiment will not be optimal for some proteins and the method is not applicable to thermally stable proteins (Refs 41, 44). Alternative models exist, for example, nonparametric analysis of response curves which compares the whole curve and provides a more robust and sensitive analysis (Ref. 42), a Bayesian semi-parametric model (Ref. 40) or a statistical scoring method for FDR-controlled analysis of 2D-TPP data (Ref. 43). Moreover, missing values are increased at higher temperature points due to complete unfolding for some proteins which can in turn affect curve quality (Ref. 43), although an isobaric trigger channel can be included to improve MS/MS analysis of peptides from these low-abundant proteins (Ref. 45).

While TPP demonstrates a bias for measuring hydrophilic soluble proteins, the addition of non-ionic or zwitterionic detergents, such as 0.4% NP40 (v/v) or 1% CHAPS (wt/v), improves the solubilisation of membrane proteins (Refs 15, 16, 46, 47, 48). Although this increases proteomic coverage (>8000 protein groups), recent research shows data indicating their destabilizing effects on the proteome. The average melting temperature across the proteome decreases upon the addition of detergent, leading to increased protein precipitation (Refs 48, 49).

Several adaptations have been proposed to enhance TPP's throughput, as a considerable limitation is the substantial number of samples required, which can be costly and labour-intensive. Additionally, the method requires expensive reagents and consumes a significant amount of mass spectrometry time. One such adaptation is the isothermal shift assay (iTSA) (Fig. 1D) which quantifies the difference in the soluble protein fraction at a single temperature (median protein *T*_m_ in the proteome under investigation). This requires only two TMT channels for direct comparison between vehicle and control sample per biological replicate, leading to a four-fold increase in throughput, allowing for more replicate analyses which improves statistical power (Ref. 50). An expected limitation of performing thermal shift assays at a single temperature is bias against proteins with higher or lower melting temperatures deviating from the median *T*_m_. Nevertheless, iTSA yielded more target identifications than TPP for staurosporine, which can likely be attributed to the increase from 2 to 5 biological replications (Ref. 50). Alternatively, to increase throughput and decrease required starting material, a one-pot method named single-tube TPP with uniform progression (STPP-UP) was recently proposed, but did not perform as well as TPP in resolution, sensitivity or accuracy (Ref. 38). Rather than subjecting aliquots of a drug or vehicle-treated sample each to different temperatures as in TPP, a single sample per treatment condition is halved, one aliquot exposed to incrementally increasing temperature for equal exposure times. Modification in protein thermostability due to ligand binding will be reflected in denaturation rate, detected as a change in relative protein abundance in the soluble fraction. The other aliquot is incubated at the low temperature for an equal incubation time, providing a baseline control to correct for differences in protein expression (Ref. 38). Ruan *et al*. explored a similar approach to iTSA, but additionally evaluated multiple concentrations at a single temperature to assess target engagement and binding affinities using label-free DIA-MS for quantitation (Ref. 51). Although these approaches require prior knowledge of the distribution of melting temperatures for the model proteome are required to determine at what temperature the largest differences can be measured.

DIA quantitation is being increasingly adopted for measuring protein solubility in thermal shift assays (Refs 51, 52). As outlined in our previous work, different label-free DIA approaches within a 1D-TPP workflow perform comparably to TMT-DDA (Ref. 53). It offers experimental flexibility with no restriction by the number of available TMT channels. Additionally, it presents a simplified sample preparation workflow as there is no labelling or fractionation, and reduced experimental costs in studies with large sample numbers (e.g. TMT reagents required for 2D-TPP) (Refs 51, 52). Furthermore, iTSA was recently coupled with a fully automated sample preparation platform (autoSISPROT) and DIA quantification to screen 20 kinase inhibitors in a fully automated manner (Ref. 54). Overall, these adapted approaches offer advantages such as simplified experimental design, shortened workflows and more statistical power as there increased number of replicates, proving sufficient for target deconvolution with increased throughput, although by summarising the curves at a fixed temperature, not as much information is gained as constructing full melting curves.

## Target deconvolution through limited proteolytic degradation

### Drug affinity responsive target stability (DARTS)

**Figure 2.**
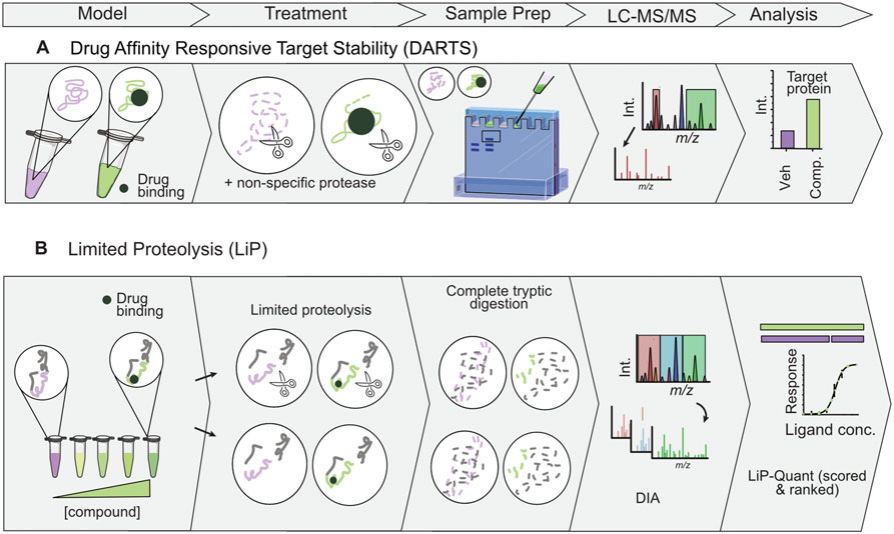
Schematic of chemoproteomic approaches based on limited proteolytic degradation. All methods are currently applicable to cell lysates only. (A) DARTS identifies proteins with enhanced resistance to proteolysis, by enriching for target proteins by SDS–PAGE. Detectable targets will have higher band intensity compared to the vehicle-treated sample. Both regions are excised for in-gel digestion and MS analysis, providing a rapid approach for target identification. (B) Lysates are treated over a concentration range and then aliquot in half. One-half is subject to limited proteolysis under native conditions, then both aliquots are subject to complete digestion under denaturing conditions, analysed with DIA. Peptides with normalised dose-dependent changes in abundance are scored using LiP-Quant score, ranking the list of targets and providing site-specific information.

Changes in protein structure following ligand-binding can alter their susceptibility to proteolysis as binding may change the access of a non-specific protease to some protein regions. This principle was applied in 2009 in drug affinity responsive target stability (DARTS) (Fig. 2A), an assay for target identification (Ref. 55). The assay can be applied in a targeted approach, where a purified form of the target protein is treated with compounds of interest or vehicle control, before being exposed to a non-specific protease for a limited time (Refs 56, 57). Unbound proteins in the vehicle-treated control that are more accessible to protease degradation will be less abundant than the drug-bound protein in the compound-treated sample. Proteolytic peptides can be removed from the remaining intact compound-bound protein by SDS–PAGE, and total intact target protein abundance measured by Western blotting, but this relies on availability of a reliable antibody (Ref. 55). Equally, this approach can be applied to probe protein targets in complex protein mixtures extracted from cells and tissues either before- (to only identifying direct targets) or after- (capture down-steam effects such as PTMs, also affecting protein structure) small molecule treatment (Ref. 55).

For discovery target deconvolution studies, after partial proteolysis, protein mixtures can be separated by 1D SDS–PAGE gel and stained (Ref. 58). The protein bands with enhanced resistance to proteolysis have a higher intensity compared to the vehicle-pronase treated sample, and so both regions are excised for in-gel digestion and mass spectrometry (Refs 55, 57). This would enable identification and relative quantification of target proteins that are negatively enriched (Refs 58, 59). Although this gel-based technique is biased towards more abundant proteins with visible differences on a gel, and ligand-bound proteins may be masked by comigrating proteins of higher abundance, consequently providing limited coverage (Refs 55, 58). To somewhat improve resolution and improve sensitivity to targets, orthogonal separation techniques were recommended such as 2D SDS–PAGE or 2D difference gel electrophoresis (2D DIGE), which are easy to implement but not very efficient (Refs 57, 58). Alternatively, after non-specific protease treatment, degraded peptides can be dialysed to provide an enriched sample of intact ligand-bound protein for bottom-up proteomic analysis, although in-gel digestion approach remains widely used (Refs 58, 60, 61, 62). A key strength of DARTS is its speed and simplicity, particularly for confirming abundant targets in complex biological samples, and not demanding large protein starting material. If treated in a stepwise manner, generating a proteolytic curve and a dose–dependence curve, the affinity of protein–ligand interactions can be estimated (Ref. 63). However, it does carry limitations in detecting low abundant proteins, and each protein has varied susceptibility to proteolysis and so the technique is not amenable to resistant or sensitive proteins (Ref. 55).

### Limited proteolysis–coupled mass spectrometry (LiP–MS)

Limited proteolysis–coupled mass spectrometry (LiP-MS) (Fig. 2B) is an alternative structural approach used to detect alterations in protein structure in complex biological matrices on a global scale by assessing enzyme accessibility to protein regions that can be altered by protein aggregation, PTM, PPI or protein–metabolite interaction (Refs 64, 65, 66, 67, 68, 69, 70, 71). For target deconvolution, lysates (to identify direct targets only), live cells (to also probe downstream targets), tissues or bodily fluids can be treated with a compound of interest, but proteins must then be extracted under non-denaturing conditions before treatment with a broad-specificity protease for a very limited time, to produce structure-specific peptides (Refs 13, 71, 72). The method requires ~10^7^ cells to yield at least 800 μg of protein and recommends four biological replicates per condition (Ref. 72). Complete tryptic digestion is subsequently performed under denaturing conditions to allow bottom-up analysis using targeted (Ref. 65) or untargeted label-free quantitation (Refs 13, 68, 69, 70). Concurrently, aliquots of the same biological extracts are digested with trypsin-only to measure total protein abundance in the samples, which are used as a normalisation factor to avoid any differences in protein abundance being interpreted as structural changes between vehicle and treated conditions (Refs 64, 68). By comparing the proteolytic patterns in drug-treated and vehicle-treated proteomes using a peptide-centric analysis, ligand-bound peptides with different normalised abundance and proteolytic pattern can be identified (Refs 65, 69, 70), using available data analysis packages (Refs 68, 73), or other software in development (Ref. 74).

Applied in 2018 to explore protein–metabolite interactions at a single-dose in microbe lysates (LiP-SMap) (Ref. 69), in 2020 the method was integrated with machine learning to tackle more complex proteomes, named LiP-Quant (Ref. 13). This reduced rates of false-positives and negatives in human cell lines by introducing dose-dependent compound treatments. Peptides with identified changes in abundance, not only correlating with a sigmoidal drug dose–response profile but also additional features identified by machine learning, are assigned a LiP-Quant score. This ranks significant targets, differentiating them from false positive identifications while providing good proteomic coverage in human cell lysates (~5200 protein groups) and cells (~6000 protein groups) (Ref. 13). However, LiP-MS was not as sensitive as TPP in detecting target engagement of kinases when tested with staurosporine, and the two approaches identified many different target proteins, suggesting complementarity when used in combination (Refs 13, 75). Sample handling adjustments were subsequently made to the protocol to increase throughput from 30 samples to 192 samples across 2 days, supporting larger studies (Ref. 68).

A notable strength of LiP-MS is its ability to provide site-specific information on binding by mapping the limited-proteolytic peptides to the protein sequence, visualised as a structural barcode (Refs 64, 68). With resolution of ~10 amino acids, compound binding sites can be identified (Ref. 68). This does require high sequence coverage, thus introduces bias to more abundant proteins, which can be somewhat improved by generating a data-dependant acquisition (DDA)-based spectral library to support data-independent acquisition (DIA) analysis, an established approach to increase proteomic coverage and quantitative accuracy (Refs 68, 69, 72). Controls can also be applied to correct for variations in proteolytic activity in the non-specific enzyme treatment, such as spiking of internal standards or endogenous peptides with known proteolytic patterns (Ref. 70). LiP-MS is not exhaustive as general extraction procedures suffer in recovery of membrane proteins, although adaptations using nondenaturing MS-compatible surfactants have shown improvement (Ref. 13). Additionally, not all compounds induce structural changes that alter the accessibility of the protein to proteolysis, resulting in false negatives (Ref. 70). Moreover, while lysis conditions are kept as physiological as possible it is essential to consider that protein extraction may induce artefactual structural alterations that are absent in live cells, potentially due to de-compartmentalization (Refs 13, 70).

## Target deconvolution approaches based on chemical denaturation

**Figure 3.**
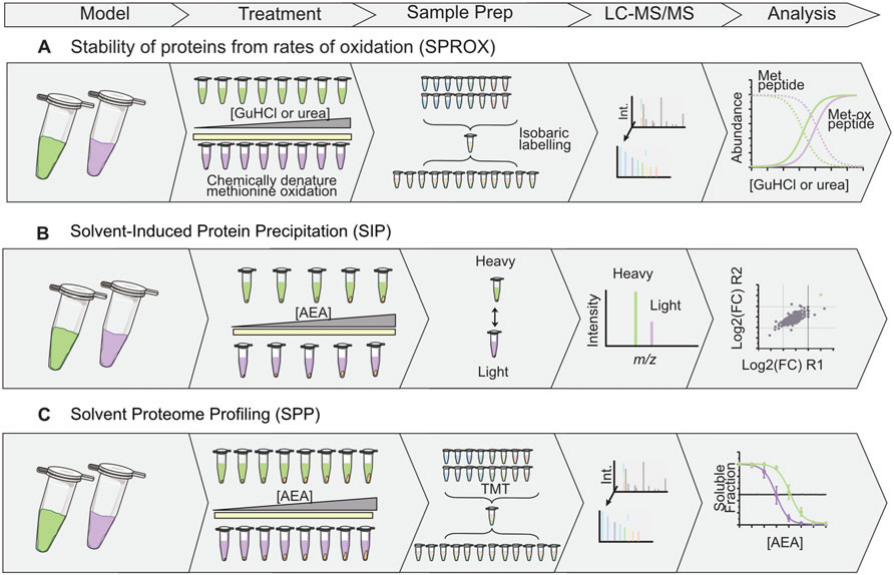
Schematic of chemoproteomic approaches based on chemical denaturation or precipitation. (A) Stability of proteins from rates of oxidation (SPROX): Cell lysates are treated with increasing concentrations of chaotropic reagent to unfold proteins, exposing methionine residues for oxidation by addition of H_2_O_2_. (B) Solvent-induced protein precipitation (SIP): Lysates are treated with increasing concentrations of solvent, causing protein precipitation. Soluble fractions are subject to dimethyl labelling, and vehicle (heavy) and treatment (light) conditions combined per concentration for LC–MS analysis. Fold change between vehicle and treated conditions are compared. (C) Solvent proteome profiling (SPP) combines the principle of SIP with TMT quantification. Each concentration is labelled, combined, fractionated and analysed to construct denaturation curves in the presence and absence of the drug to calculate shift in the concentration of solvent at which 50% of the protein is unfolded.

Chemical stress can be used to destabilise proteins, allowing assessment of their differential stability caused as a result ligand binding. For example, chaotropic reagents disrupt hydrogen bonds and induce protein unfolding (Ref. 76). Stability of proteins from rates of oxidation (SPROX) (Fig. 3A) uses this to measure the stability of proteins by exposing compound or vehicle treated lysate sample to a concentration series of chemical denaturing conditions, such as guanidine hydrochloride or urea (Ref. 77). They are incubated with equal amounts of hydrogen peroxide to oxidise the accessible methionine side chain residues to assess the thermodynamic properties of proteins and protein–ligand complexes (Refs 77, 78). The binding of a compound to proteins causes changes in their propensity to denature, resulting in the requirement of different amounts of denaturing agent to unfold and expose methionine residues. Non-oxidised and oxidised peptides can be measured using various quantitative workflows such as chemical labelling (Ref. 79). This method is reliant on quantifying methionine-containing peptides and can require substantial amounts of starting material for protein lysates (2–3 mg) (Refs 79, 80, 81). Another method called pulse proteolysis (PP) is based on a similar principle to SPROX by firstly treating proteins with denaturing chemicals, but susceptibility to proteolysis in presence of ligand is measured instead of methionine oxidation. The protein degradation curve will be shifted if a compound has engaged a protein target (Ref. 82).

Chemical denaturation by solvent and acidic stresses can be monitored by measuring protein solubility (Refs 83, 84). Lysates can be treated with increasing concentrations of organic solvent or acid causing protein unfolding and aggregation, and the soluble protein fraction quantified between treatment and vehicle conditions. For example, solvent-induced protein precipitation (SIP) (Fig. 3B) uses increasing percentage of an organic solvent mixture (acetone/ethanol/acetic acid at a 50:50:0.1 ratio) after a single drug dose, or a dose–response at a single percentage of organic solvent to assess affinity drug–target interaction (Ref. 84). Dimethyl labelling with single-shot LC–MS/MS analysis is used for quantitation but does provide relatively low proteomic coverage (1854 proteins) compared to TPP studies that utilise TMT and fractionation (Ref. 84). Solvent proteome profiling (SPP) (Fig. 3C) uses TMT and fractionation to generate full denaturation curves, similar to TPP and identified > 7600 proteins in two biological replicates (Ref. 85).

## Target deconvolution approaches based on protein-solubility in cells

Common across the various methods discussed, is the measure for differences in protein (or peptide) abundance within the soluble fraction post-exposure to denaturing or degrading factors such as temperature (Ref. 22), chemical (Refs 84, 85, 86, 87), or proteasomal (Refs 55, 69). To address some of the drawbacks inherent in these approaches, particularly low-throughput, proteome integral solubility alteration (PISA) assay was developed to measure variations in solubility across the proteome, while reducing analysis time and sample consumption (Ref. 88).

**Figure 4.**
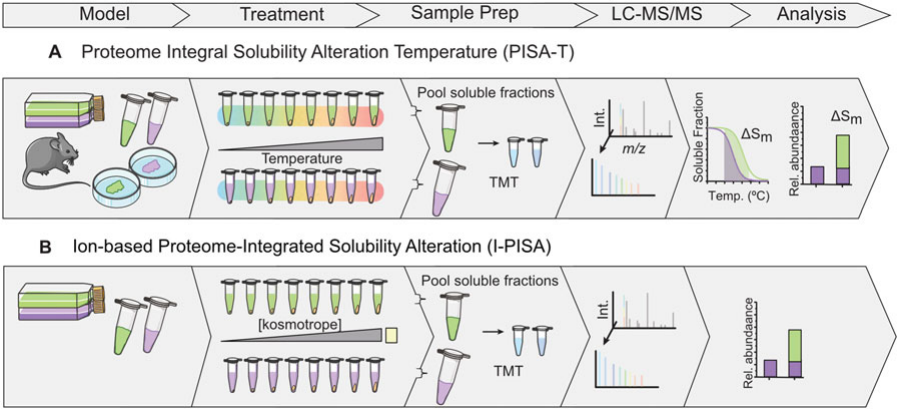
Schematic of chemoproteomic approaches measuring alterations in solubility, applicable to live cells and lysates. (A) Proteome integral solubility alteration – temperature (PISA-T): utilises a temperature-centric approach, pooling soluble fractions across temperature points prior to TMT labelling and fractionation, to measure protein abundance and difference in area under the melting curve. A 2D format is optional for added specificity. (B) PISA-I, measures protein solubility in live cells post-denaturation using a kosmotropic gradient. Similarly, soluble fractions are pooled prior to TMT labelling followed by fractionation. An advantage is that more TMT channels are available compared with TPP, allowing more samples to be compared quantitatively in a single experiment.

In 2019, this assay was first applied in a temperature-centric approach, termed PISA-T (Fig. 4A) and was benchmarked with thermal proteome profiling showing notable correlation (Ref. 88). The workflow maximises the number of samples analysed within a single TMT batch, by pooling the soluble fractions across temperature points before TMT labelling. Measuring protein abundance (Sm) in each consolidated sample, which represents the total area under its melting curve, regardless of curve shape thus overcoming challenges in sigmoidal curve fitting faced with traditional TPP (Refs 42, 88). Subsequent calculation of differences between areas under the melting curves or fold changes (ratios between integral samples) signify ligand-induced alterations in protein solubility because of direct compound binding in cell lysates, or potentially due to perturbations in associated complexes or PTMs when performed in live cells (Refs 88, 89, 90). The assay was also performed in a 2D format, introducing a third sample/channel per biological replicate, which is a consolidation of a concentration-dependant curve, providing added specificity (Ref. 89). Addition of mild detergent such as 0.4% NP-40 was included in recent protocols to improve proteomic coverage (Ref. 91).

A significant advantage of PISA is the ability to perform many biological replicates within a single TMT-plex, providing higher statistical power for protein solubility shifts (Ref. 90) especially when utilising 16- or 18-plex TMTpro (Ref. 91). Such datasets provide excellent proteomic coverage (>8000 proteins) with little missing values (Ref. 92), that are a challenge when performing multi-batch TMT required in classical thermal proteome profiling (Ref. 93). An untreated sample can also be analysed as a carrier proteome in a single TMT channel for improving detection and quantification of less abundant peptides in PISA samples (Ref. 88). The increased throughput of PISA provides flexibility for experimental design and has allowed for many adaptations of the workflow, allowing more complex assessment such as comparison of multiple compounds (Ref. 94). Other examples are PISA-express, which allows simultaneous assessment of protein expression and thermal stability, used to study pluripotency in stem cells (Ref. 95), antibiotic mode of action in bacteria (Ref. 96), and PISA-REX to additionally analyse redox (Ref. 97). Moreover, residence time PISA (ResT-PISA) introduced a temporal perspective of each drug–target complex by measuring protein solubility profiles after drug treatment removal in cell lysates (by filtering) or intact cells (by cell washing) providing a unique value in supporting the prediction of *in vivo* drug efficacy (Ref. 98).

PISA assay has also been applied to measure protein solubility following denaturation by stepwise treatment with kosmotropic reagents (PISA-I) (Ref. 86) or solvents (solvent-PISA) (Ref. 85), continuing area under the curve analysis. PISA-I applies to live cells (Fig. 4B) and introduces an additional step of quenching kosmotropic ions resulting in an insoluble salt prior to pooling. Known drug targets (in lysates) and downstream effectors in living cells were identified. Notably, some proteins detected by I-PISA had unique and opposing behaviour in solubility when compared to T-PISA in cell lysates, highlighting the value of using complementary denaturation conditions to probe protein-small molecule interactions. Known targets were also successfully identified from 1 μg (equivalent to ~7000 cells) of compound treated lysate among 691 quantified proteins, providing immense value when there are constraints associated with the initial quantity of cellular material (Ref. 86).

## Concluding remarks

To summarise, each method presents distinct advantages for studying protein–ligand interaction, offering insights at either the protein or peptide level under various biological contexts. Collectively, they enable discovery of compound targets, off-targets and structural information on specific binding sites on a proteome-wide scale. All of which have significantly advanced target deconvolution, overcoming previous limitations through enhanced mass spectrometry sensitivity and improved quantitative workflows. Ongoing challenges related to throughput are being addressed by automated sample handling, pooling of soluble fractions to support large-scale studies. Trends in method development emphasise the use of carrier proteomes and isobaric trigger channels to improve sensitivity, particularly as we navigate towards low-sample scenarios. Additionally, incorporating proteomic samples in experiments for normalisation has proven beneficial in resolving and identifying more targets, enhancing sensitivity for ligand-induced alterations. As quantitative workflows continue to evolve, whether through enhanced instrument resolution, advancements in TMTpro technology, or the capabilities of DIA, chemoproteomic abilities will continue to accelerate drug discovery.
